# Influence of perioperative complication severity on 1- and 2-year outcomes of low back surgery

**DOI:** 10.1007/s10195-016-0436-5

**Published:** 2016-11-22

**Authors:** James Grainger, Thomas Hammett, Robert Isaacs, Chad Cook

**Affiliations:** 1 Department of Orthopedics, Duke University School of Medicine, 2200 W Main St, Durham, NC 27708 USA; 2 Division of Neurosurgery, Duke University Medical Center, 200 Trent Drive #1l, Durham, NC 27710 USA; 3 Department of Orthopedic Surgery, Duke University, 2200 W. Main St. B230, Durham, NC 27708 USA

**Keywords:** Perioperative complications, Low back surgery, Lumbar spine, Outcomes

## Abstract

**Background:**

Several factors potentially influence outcomes of surgery, including perioperative complications. Complications may take many forms and the Clavien–Dindo (CD) classification is designed to categorize them by degree of severity. The aim of this study was to evaluate the influence of perioperative complications by severity categorization on the 1-and 2-year pain and disability outcomes for patients who received low back surgery.

**Materials and methods:**

Data used for the study involved a purposive sample (*N* = 477; 8.1%) from a spine outcomes registry of 5876 patients who received spine surgery and encountered complications. All complications were categorized using the CD classification and were collapsed according to distribution frequencies, i.e., Grade I–II and Grade III–V. Adjusted and unadjusted regression analyses were used to determine the association between CD classification and 1- and 2-year outcomes.

**Results:**

The majority of surgical complications were Grade III−V (*N* = 358; 75.1%), with two incidences in which death occurred. For the unadjusted models, there were no significant associations between CD classification categorizations for 1-year outcomes; however, 2-year outcomes were significantly worse (*P* <0.05) for those with Grade III–V categorization. When adjusted and controlled for baseline characteristics, CD classification did not influence 1-or 2-year pain and disability outcomes.

**Conclusions:**

When control variables are considered, the severity of perioperative surgical complications does not appear to influence 1- or 2-year pain and disability outcomes.

**Level of evidence:**

Level 4.

## Introduction

Spine surgery is a relatively common procedure in the United States. One form, spinal fusion, has rapidly increased in prevalence by 220% between 1990 and 2001 [1]. According to the Agency for Healthcare Research and Quality (AHRQ), approximately 427,000 spinal fusions and 417,000 laminectomy surgeries were performed in the United States in 2013 [2].

Complications are an unfortunate consequence of surgery and may influence recovery rates, quality of life and healthcare costs [3]. They may take the form of wound-related, surgical or medical complications [4]. The National Inpatient Sample showed an overall complication incidence of 13.1% from 1993 to 2002 [4]. Others have reported variable figures depending on the type of surgery, with cervical spine surgery (8.9%) exhibiting fewer complications than thoracolumbar surgery (17.8%) [5]. Complications are the most frequent reason for hospital readmissions [3], with the AHRQ reporting readmission rates of 6.4% for fusions, and 6.5% for laminectomy [2].

Surgical complications have varying levels of severity, with some causing only minor symptoms and others leading to severe disability or even death [6]. Although there are several complication indexes that exist, there are no specific tools that are advocated by clinical practice guidelines. One existing tool is the Clavien*–*Dindo (CD) classification system [7] (Table 1). The CD classification system separates surgical complications into five grades based on the treatment required to correct the complication.

**Table 1:** Clavien–Dindo classification description and frequency of surgical complications as reported by spine surgeons

Care required	Complication	Total reported
Grade I–II Clavien–Dindo		
Does not require alterations in the postoperative course of treatment, or are without need for additional pharmacological, surgical, endoscopic, or radiological interventions to treat the complication itself	Adjacent level disease/disc herniation—minor [22]	0
Requires pharmacological treatment with drugs, including blood transfusions and total parenteral nutrition	Cardio/pulmonary [23]	71
	DVT/vascular/embolism [24]	8
	Fracture—minor medical [25]	0
	Fracture—minor surgical [25]	0
	GI/GU—minor medical [26]	26
	GI/GU—minor surgical [26]	0
	Medical infection—minor [27]	13
	Wound infection—minor [28]	13
Grade III–IV Clavien–Dindo		
Requires surgical, endoscopic, or radiological interventions to be corrected (III) or life-threatening single/multi-organ dysfunctions that require ICU management (IV)	Adjacent level disease/disc herniation—major [29]	0
	Bleeding/transfusion [30]	55
	CSF/dural tear [31]	261
	Fracture—major medical [25]	0
	Fracture—major surgical [25]	0
	GI/GU—major medical [26]	2
	GI/GU—major surgical [26]	3
	Hardware [32]	41
	Medical infection—major [33]	1
	Neural [34]	19
	Nonunion [35]	1
	Wound infection—major [28]	0
Grade V Clavien–Dindo		
Results in death of the patient	Death	2

To our knowledge, two studies have explored the role of complication severity on outcomes [8, 9]. Glassman et al. [8] explored the influence of major and minor perioperative complications of spine surgery on 1-year disability, pain, and quality of life outcomes and found that major complications, although rare, negatively influenced quality of life. Fritzell et al. [9] compared complications among three different types of fusion surgery and found no significant differences between complications ranked major or minor and 2-year outcomes [9]. Both studies involved small sample sizes of individuals with complications (<100). Our goal was to explore the relationship between severity (by rank) of perioperative outcomes with disability and pain outcomes at both 1- and 2-year time frames in a larger sample of patients who experienced complications. We hypothesized that those with higher categorization according to the CD classification would exhibit poorer outcomes for disability and pain [7].

## Materials and methods

### Study design

This was a retrospective secondary database analysis that used data obtained from a multi-institutional, prospective spine outcomes registry (Prostos). The full spine outcomes registry involved data compiled from 14 spine surgical institutions in two countries (United States and Canada), and incorporated surgical results from 40 medical physicians who specialized in spine surgery. Individuals within the registry were recipients of multiple forms of thoracolumbar surgery including discectomy, fusion, decompression with discectomy, or decompression with fusion. Data from the registry have been used previously in two distinct studies [10, 11].

### Participants

Participants for this study involved patients with lumbar disorders who received lumbar surgery between 2002 and 2012. There were no restrictions on type of diagnosis, type of surgical fusion approach, or age. To meet the objective of this study (associate complication severity to outcomes), data were only gathered on individuals who had one or more perioperative complications and who had reported year 1- and 2-year outcomes. In total, 5876 patients were screened to identify the presence of perioperative complications (*N* = 478; 8.1%). Examples of perioperative complications included wound, neural, deep vein thrombosis, reoperation, and a number of other forms (Table 1).

### Clavien–Dindo classification/predictor variable

Our goal was to appropriately rank the severity of perioperative complications, so we therefore converted the narrative of each documented complication (*N* = 478) to a CD classification grade (I−V). Grade I surgical complications do not require alterations in the postoperative course of treatment, or are without the need for additional pharmacological, surgical, endoscopic, or radiological interventions to treat the complication itself. Grade II complications require pharmacological treatment, including blood transfusions and total parenteral nutrition. Grade III complications require surgical, endoscopic, or radiological interventions to be remediated. Grade IV complications are life-threatening single/multi-organ dysfunction(s) and require ICU management. Grade V complications result in death of the patient. Patients with multiple complications of different CD rankings were classified by the highest grade of complication. For example, a patient with a minor medical infection (Grade II) and a dural tear (Grade III) would be classified as a patient with a Grade III complication.

Interestingly, none of the 478 narratives qualified as a Grade I CD classification and only two were categorized as Grade V (death). By far, the majority of the perioperative complications reported in the database were Grade II and Grade III. Due to the disproportional inter-classification frequencies that were found, Grade I and II complications were collapsed into a single group, as were Grades III and IV. The CD classification system was designed to allow for the combination of groups in order to simplify its use depending on the patient cohort being analyzed [12]. Since the study involved investigating pain and disability outcomes at 1 and 2 years, Grade V complications were removed from the study. Classification distributions are shown in Table 2.

**Table 2:** Patient Clavien–Dindo distribution

Grade	Number of patients
I–II	119
III–IV	356
V	2 (removed from study)

### Control variables

Study variables included (1) age, (2) body mass index (BMI), (3) gender, (4) previous back surgery history, (5) baseline Oswestry Disability Index (ODI), (6) unique baseline visual analog scale for pain (VAS) for the low back, (7) baseline SF-12 physical component summary (PCS) scores, (8) baseline SF-12 mental component summary (MCS) scores, and (9) number of spinal levels treated during surgery. The SF-12 MCS scores and SF-12 PCS scores reflected the subscales for the SF-12 Quality of Life questionnaire, which is routinely used in clinical practice for assessment of spine surgery [13].

### Outcomes measures

For this study, two different outcome measures were used—(a) percent change in pain (VAS) and (b) percent change in disability (ODI). Percentage change for pain and disability was calculated by taking the difference of the VAS for pain and the ODI score (from baseline to the 1-year follow-up), and dividing the difference by the baseline score, and then multiplying by 100. The end product was a positive or negative percentage change expressed as a whole number. Use of percent change and the inclusion of a minimum of two different outcome constructs have been recommended by the Initiative on Methods, Measurement, and Pain Assessment in Clinical Trials (IMMPACT) group [14].

The IMMPACT work group [13] recognized a 30% reduction in pain and disability from baseline as a minimally clinically important threshold of success [15]. Subsequently, we dichotomized outcomes into two groups—(1) those with <30% reduction in pain and disability and (2) those with ≥30% reduction in pain and disability, for 1- and 2-year outcomes. These variables served as the outcome (dependent) variables for the study.

### Determining appropriate number of observations per variable

For simple univariate multinomial or logistic regression, Hosmer and Lemeshow [15] recommended a minimum observation-to-variable ratio of 10, but cautioned that a number this low will likely over-fit a model. Nevertheless, we adopted their preferred observation-to-variable ratio of 20:1 for the multivariate modeling, thus minimizing our maximum independent variables to <20. With only one predictor variable and potentially eight control variables, we estimated that there was very little risk of over-fitting our model.

### Data analysis

All analyses were performed using Statistical Package for the Social Sciences, version 20.0 (SPSS Inc., Chicago, IL, USA). Baseline characteristics were plotted by means and standard deviations or by frequencies for the 30% outcomes thresholds (minimally clinically important change) for both VAS and ODI. Each complication was divided by CD classification of high (Grades III–IV) or low (Grades I–II) [7]. SPSS was instructed to ignore missing values since data were not available on outcomes measures at 1 and 2 years in >20% of the sample. Univariate and multivariate (adjusted) logistic regression analyses were performed using the CD classification as the predictor in each model (ODI 30%, VAS 30%) [15]. For the adjusted models, all variables that were found to be significantly different during baseline bivariate comparisons were used as control variables. For all analyses, individual *P* values, odds ratios (OR) and 95% confidence intervals (CI) were reported. For all models, a *P* value of <0.05 was considered significant.

## Results

The complication grades were I/II (*N* = 120) and III/IV (*N* = 356) (Table 3). Follow-up ODI scores were collected in 62.1% of patients at 1 year, and 35.8% at 2 years. The follow-up rates for the VAS at 1 and 2 years were 58.9 and 35.8%, respectively. When comparing high and low complication categories, statistically significant differences were present for number of levels of surgery addressed (2.7 vs 1.6), higher levels of reported baseline disability, and lower baseline levels of reported quality of life (both MCS and PCS).

**Table 3:** Descriptive baseline comparisons of high (I–II) and low (III–IV) complication groups by Clavien–Dindo classification

	Mean (SD)/frequency/category for CD Grade I–II	Mean (SD)/frequency/category for CD Grade III–IV	*P* value
Age	58.3 (13.9)	60.0 (12.9)	0.26
Gender	53 F 42 M 24 Not reported	215 F 139 M 12 Not reported	0.38
Body mass index	28.8 (6.3)	29.8 (6.2)	0.21
Baseline pain score	7.1 (2.5)	7.2 (2.4)	0.58
Baseline ODI score	47.9 (16.4)	51.4 (13.6)	**0.04**
Baseline SF-36 PCS score	30.7 (8.9)	27.6 (6.4)	**<0.01**
Baseline SF-36 MCS score	41.7 (12.3)	37.7 (14.1)	**<0.01**
Levels of surgery	1.6 (1.0)	2.7 (1.3)	**<0.01**
Number of patients with prior surgery	29 Y 90 N	73 Y 285 N	0.36
1-year VAS percent change	25 Y 23 N	123 Y 110 N	0.92
2-year VAS percent change	18 Y 8 N	75 Y 72 N	**0.03**
1-year ODI percent change	25 Y 26 N	121 Y 124 N	0.96
2-year ODI percent change	21 Y 13 N	78 Y 103 N	**0.04**

Bold numbers indicates significant difference

*F* female, *M* male, *N* no, *Y* yes

Unadjusted univariate regression analyses suggest that lower levels of CD classification were significantly associated with increased improvement at final outcome (2 years) in both pain (OR 2.88; 95% CI 1.08, 7.66) and disability (OR 2.13; 95% CI 1.01, 4.52), a finding that was not significant at 1 year. In patients who experienced Grade I−II complications, there was significantly increased improvement in pain and outcome compared to patients who experienced Grade III–IV complications.

When gender, levels of surgery, baseline disability, and quality of life (MCS and PCS) were used as controls, the adjusted multivariate analyses suggest that CD classification does not contribute to pain and disability outcomes at 1 or 2 years. Table 4 provides the findings of univariate and multivariate regression analyses.

**Table 4:** Comparative analyses of disability and pain by Clavien−Dindo classification at 1 and 2 years (30% difference)

Variables	Odds ratio (95% confidence interval)	*P* value
Unadjusted oswestry disability score		
Grade I–II Clavien–Dindo classification (year 1)	0.98 (0.54, 1.80)	0.96
Grade I–II Clavien–Dindo classification (year 2)	2.13 (1.01, 4.52)	**0.04**
Adjusted Oswestry Disability Score^a^		
Grade I–II Clavien–Dindo classification (year 1)	1.13 (0.57, 2.21)	0.72
Grade I–II Clavien–Dindo classification (year 2)	1.74 (0.73, 4.19)	0.21
Unadjusted visual analog scale for pain		
Grade I–II Clavien–Dindo classification (year 1)	0.97 (0.52, 1.81)	0.93
Grade I–II Clavien–Dindo classification (year 2)	2.88 (1.08, 7.66)	**0.03**
Adjusted visual analog scale for pain^a^		
Grade I–II Clavien–Dindo classification (year 1)	0.91 (0.45, 1.82)	0.78
Grade I–II Clavien–Dindo classification (year 2)	2.23 (0.78, 6.36)	0.13

Bold numbers indicates significant difference

^a^Control variables include gender, number of spinal levels, disability score at baseline, and SF-36 physical and mental component summary scores

## Discussion

The aim of this study was to determine the relationship between the severity of perioperative complications and 1- and 2-year outcomes using the ODI and VAS. During the perioperative phase, the severity of complications can vary significantly, which necessitates the use of a classification system to rank complications based on severity when relating complications to outcomes. Furthermore, perioperative complications are usually medical or surgical-based and the long-term relationship between these variables and patient outcomes is unexplored. We used the CD classification system, which ranks severity into five ordinal categories based on the therapeutic intervention used to manage the complication [7]. Our findings suggest no significant relationship between the severities of perioperative complications at 1 year; however, at 2 years those in the Grade III–IV category (higher severity) had significantly worse outcomes in both the ODI and VAS in an unadjusted model. However, when controlled for baseline characteristics, there were no significant relationships between the two groups at either 1 or 2 years with each of the outcome measures. There are a number of potential reasons for these findings.

The most common spine surgery complication requiring readmission is wound infection of the surgical site [3]. Wound infections most commonly manifest around 13 days, long after the individual has been discharged from the hospital or ambulatory care center [16]. We studied perioperative complications and the most commonly seen were cerebrospinal fluid leakage and dural tears, cardiac/pulmonary related, and bleeding/transfusions. We hypothesized that the severity of complications identified in the study would influence long-term morbidity (pain and disability). Contrary to our hypothesis, the baseline characteristics of the individual influenced outcomes more than the perioperative complication severity of the individuals when adjusted in the modeling.

As stated, other factors may influence outcomes more than complications. Baseline differences were present among those in the low and higher severity groups for a number of spinal levels of surgery, quality of life scores, and disability scores. Operating on more spinal levels increases risk for injury in a greater number of neighboring structures, and performing surgery at specific levels may lead to a greater risk for serious complication [17]. Benedetti-Valentini et al. explained this in regard to performing disc surgery and laminectomy at L1-L2, where there is a risk for perforation of the abdominal aorta and inferior vena cava based on location. Psychological factors such as depression and poor SF-36 MCS scores (which measures emotional health, vitality, social and general health perceptions) are associated with poorer outcomes [14]. Non-favorable outcomes have also been associated with catastrophizing and neuroticism at baseline [14].

Although not formally investigated, we feel there is a chance that the baseline health status of the individuals in the higher CD classification reflected their poorer disability levels (captured with the ODI) and could have predisposed the individuals to greater complications. For example, within the literature diabetes is affiliated with an increased risk of infection during spinal surgery as is congestive heart failure, peripheral vascular disease, cerebrovascular disease, dementia, rheumatic disease, peptic ulcer, liver disease, hemiplegia, renal disease, malignancy, liver disease, metastasis, thrombophlebitis, fracture, alcoholism, scar, DVT, burns, dehydration and malnutrition [18]. Certainly, requiring surgery to more levels complicates the process and increases the likelihood of bleeding, dural incision, etc. [19, 20].

Another factor may be the outcome measures that were used in our study. In a 2003 study, Fritzell et al. [9] examined the relationship between complications experienced during lumbar fusion surgery and outcome measures. They compared those with complications to those without, and found no significant association between complications and 2-year outcomes using the ODI and VAS. However, their study also included a Global Assessment of Treatment question at 2 years post operation where patients were asked if they were currently ‘much better’, ‘better’, ‘unchanged’, or ‘worse’ than before surgery. It should be noted that the Global Assessment of Treatment scale used in their study has not been validated, and the results should therefore be considered with some degree of apprehension. Although the finding lacked statistical significance (*P* = 0.052) there was a trend toward patients who experienced complications to be less likely to reply ‘much better’ than those who were free of complications [10]. This suggests that different outcome tools may lead to differences in results. It has been recommended that at least five different outcomes measures should be used for lumbar spine assessment of recovery, assessing the following five domains—back-specific function, health status, pain, work disability, and patient satisfaction [21].

Our findings are very similar to a study by Glassman et al. [8] who reported minimal differences in outcomes at 1 year when comparing those with no complications, minor complications and major complications [9]. In this study by Glassman and colleagues, complications were classified as major or minor by a consensus agreement between the participating surgeons. The outcome measures considered were the SF-12 PCS, the SF-12 MCS, SF-12 domain-specific subscale, the ODI, the numeric rating score for back and neck pain, and the SRS-22 total score and subscale scores. There were no significant differences found between the complication groups in any of the utilized outcome measure scores except for the SF-12 general health subscale, where the score of the major complication group was significantly worse than the minor complication group. The score of the group with no complications was not significantly different than either of the other two groups.

### Limitations

Although the present study utilized a sample size larger than any known previous studies on this subject, it not sufficiently comprehensive to conclusively delineate the long-term risk associated with the severity of perioperative complications experienced by all patients undergoing lumbar spine surgery. A larger sample size that encompasses a more diverse patient population in terms of baseline characteristics and primary conditions would improve the study’s external validity. Furthermore, the follow-up rates of this study were of a smaller percentage than those typically seen in prospective designs or trials. Another limitation of the present study was the varying number of techniques used for surgical correction. The database included inputs for anterior lumbar interbody fusion, posterior lumbar interbody fusion, extreme lumbar interbody fusion, transforaminal lumbar interbody fusion, posterolateral fusion, posterior posterolateral fusion, and options for open access or minimally invasive techniques. While it was important to capture as many surgical outcomes as possible, we did not control for the risks associated with different types of surgical techniques.

## Appendix 1: Description of complications

**Table:**

Grade I Clavien–Dindo	
Adjacent level disease/disc herniation—minor [22]	Symptomatic deterioration of spinal levels adjacent to the site of a previous fusion, often leading to disc herniation. Disease did not require surgical treatment
Grade II Clavien–Dindo	
Cardio/pulmonary [23]	Cardiovascular complications include myocardial infarction and congestive heart failure. Pulmonary complications include atelectasis, pneumonia, pleural effusion and respiratory failure
DVT/vascular/embolism [24]	Blood clot that forms in the deep veins of the body, usually in the lower extremity. When the thrombus breaks away from the vessel wall and travels in the blood stream, it is considered an embolism. DVTs can be confirmed with a venogram, computed tomography scan, magnetic resonance imaging, or pathological examination of thrombus removed during surgery or autopsy
Fracture—minor medical [25]	One or more bones are broken perioperatively but not as a direct result of the operation. Minor medical fractures did not require surgical intervention for correction
Fracture—minor surgical [25]	One or more bones are broken perioperatively as a direct result of the operation. Major surgical fractures required surgical intervention for correction
GI/GU—minor medical [26]	Any disturbance of the gastrointenstinal and or genitourinary system not requiring surgical correction and not due to unintentional surgical error
GI/GU—minor surgical [26]	Any disturbance of the gastrointestinal and/or genitourinary system not requiring surgical correction and that resulted from unintentional surgical error
Medical infection—minor [27]	Medical infection is an infection not specifically derived from the wound created from surgical incision. Minor infection does not require surgical correction, but may require treatment with antibiotics
Wound infection—minor [28]	Perioperative wound infection is most commonly a result of contamination of the surgical wound during surgery. Minor infection is usually superficial in nature and does not require surgical correction, but may require treatment with antibiotics
Grade III Clavien–Dindo	
Adjacent level disease/disc herniation—major [29]	Symptomatic deterioration of spinal levels adjacent to the site of a previous fusion, often leading to disc herniation. Disease required surgical treatment
Bleeding/transfusion [30]	The procedure results in sufficient blood loss to necessitate a blood transfusion
CSF/dural tear [31]	Unintentional dural tear as a result of surgical error. Dural tears cause cerebral spinal fluid (CSF) to leak and are often correlated with lumbar burst fractures
Fracture—major medical [25]	One or more bones are broken perioperatively, but not as a direct result of the operation. Major medical fractures required surgical intervention for correction
Fracture—major surgical [25]	One or more bones are broken perioperatively as a direct result of the operation. Major surgical fractures required surgical intervention for correction
GI/GU—major medical [26]	Any disturbance of the gastrointestinal and/or genitourinary system requiring surgical correction and not due to unintentional surgical error
GI/GU—major surgical [26]	Any disturbance of the gastrointestinal and/or genitourinary system requiring surgical correction that resulted from unintentional surgical error
Hardware [32]	Metal plates, rods, screws or other implanted devices break and/or move from the correct placement before the tissue has sufficiently healed
Medical infection—major [33]	Medical infection is an infection not specifically derived from the wound created from surgical incision. Major infection requires surgical correction
Neural [34]	Damage to the central or peripheral nervous system
Nonunion [35]	A failed spinal fusion in which the segments of vertebral bone do not merge over the disc space
Wound infection—major [28]	Perioperative wound infection is most commonly a result of contamination of the surgical wound during surgery. Major infection may be deep or cause organ infection and requires surgical correction
Grade IV Clavien–Dindo	
Death	Procedure results in mortality
